# A facet atlas: Visualizing networks that describe the blends, cores, and peripheries of personality structure

**DOI:** 10.1371/journal.pone.0236893

**Published:** 2020-07-30

**Authors:** Ted Schwaba, Mijke Rhemtulla, Christopher J. Hopwood, Wiebke Bleidorn

**Affiliations:** Department of Psychology, University of California, Davis, Davis, California, United States of America; Aalborg University, DENMARK

## Abstract

We created a facet atlas that maps the interrelations between facet scales from 13 hierarchical personality inventories to provide a practically useful, transtheoretical description of lower-level personality traits. We generated this atlas by estimating a series of network models that visualize the correlations among 268 facet scales administered to the Eugene-Springfield Community Sample (Ns = 571–948). As expected, most facets contained a blend of content from multiple Big Five domains and were part of multiple Big Five networks. We identified core and peripheral facets for each Big Five domain. Results from this study resolve some inconsistencies in facet placement across instruments and highlight the complexity of personality structure relative to the constraints of traditional hierarchical models that impose simple structure. This facet atlas (also available as an online point-and-click app at tedschwaba.shinyapps.io/appdata/) provides a guide for researchers who wish to measure a domain with a limited set of facets as well as information about the core and periphery of each personality domain. To illustrate the value of a facet atlas in applied and theoretical settings, we examined the network structure of scales measuring impulsivity and tested structural hypotheses from the Big Five Aspect Scales inventory.

## Introduction

Over the past few decades, a general consensus has emerged regarding the structure of individual differences in higher order personality traits [1]. In this hierarchical model, two superordinate factors (alpha and beta) subsume five broad domains (the Big Five; extraversion, agreeableness, conscientiousness, neuroticism, and openness to experience), which can be subdivided into narrower facets and even narrower nuances [2–4]. Similar hierarchical models have been developed for personality pathology [5,6] and mental disorders more generally [7].

Although the field has come to a general agreement concerning the number and content of factors at the higher levels of the hierarchy (but see [8]), there is very little consensus regarding the lower-level structure of facets [9–11] (for the purposes of this paper, we define a personality facet as any personality trait that is narrower than a domain yet broader than a specific behavioral nuance). Common lower-order measures range from as few as 10 lower-order facets [12] to as many as 45 [13]. Some of these measures were developed specifically to measure the spaces below each of the Big Five domains (e.g. [10]), whereas other measures subdivide the facet space in unique ways that do not correspond closely to the Big Five (e.g. [14, 15]). There is also little agreement about how to name these facets. This leaves room for jingle fallacies in which facet scales with similar labels measure different constructs, as well as jangle fallacies, in which differently-labeled facets measure the same construct [16]. Together, these issues have complicated the development of both personality tests and theory [17].

Faced with similar problems, the field of genetics has created atlases that allow researchers to easily identify a particular gene’s chromosomal region, function, and co-occurrence with other genes (e.g., [18]). These atlases help researchers understand individual genes, facilitate communication between scholars, and improve the coherence of research programs. Atlases have similarly been used in clinical personality assessment to standardize interpretation of test scores across clinicians (e.g., [19]).

We believe the idea of an atlas can be fruitfully applied to the study of personality facets. A facet atlas could be used as a practical reference guide for researchers or clinicians with personality data or as an investigative resource for researchers exploring questions regarding facet structure. We accordingly created a facet atlas that summarizes the interrelationships between existing facet scales and the associations between facets and the Big Five domains. To do this, we estimated a series of network models that visualize and summarize the patterns of correlations between 268 facet scales from 13 hierarchical personality measures administered to the Eugene-Springfield Community Sample (ESCS; see [20]). We addressed three questions to demonstrate the value of this facet atlas for personality assessment and theory. First, to what extent are facets *blended* representations of multiple Big Five domains? Second, which facets compose the *core and periphery* of each Big Five domain? Third, how can this facet atlas be used to better understand *particular constructs and instruments*?

### Blended facets

Personality traits do not have a simple structure [21,22]. Even measures designed to maximize each facet’s correspondence with a single domain often contain facets that have substantial associations with multiple domains [21]. For example, although interpersonal warmth is classified as a facet of extraversion in some measures and as a facet of agreeableness in others [10], it is a blend of both agreeableness and extraversion [23]. As such, the placement of warmth within one or the other of these domains is somewhat arbitrary.

Some domains form a circumplex, where common variance between the two is occupied by meaningful traits [13]. This has been well-documented for extraversion (agency) and agreeableness (communion), the domains that organize the interpersonal circumplex [23–25]. By capturing all blends of these two domains, this model has allowed interpersonal researchers to identify evidenced-based principles for how dyads interact [26,27] and has provided interpersonal clinicians with a useful rubric for case formulations about individual patients [28,29].

One past effort sought to create a circumplicial periodic table of blended facets, using 8 questionnaires assessed in the same dataset as the present study and two questionnaires assessed in a different sample [30]. Woods and Anderson (2016) identified 22 common blends and provided a term for each (for example, high conscientiousness and low extraversion was termed cautiousness). Notably, although they found content to represent most Big Five blends, they found very little content measuring either positive blends or contrasts of agreeableness and conscientiousness.

In the present study, we investigate if this finding holds when additional facets are included from other hierarchical personality inventories, and we investigate whether some facets contain blends of three or more Big Five domains. Indeed, some areas between higher-order domains that are currently not well measured. These spaces may be unmeasured because of test development biases favoring simple structure, and improving coverage of these spaces might enhance the comprehensiveness of personality trait measurement. Alternatively, these uncommon blends might reflect necessarily empty space where personality variation largely does not exist [31]. This interpretation would spur research into the reasons *why* these spaces are empty (e.g., it may be that few behaviors relate simultaneously to both domains).

### Domain cores and peripheries

Not all personality facets relate equally to their parent domain. Some facets are situated conceptually and empirically at a domain’s core, as indicated by strong correlations with many other facets within that domain. Imagination, for example, is a core facet of openness that is correlated with most other openness facets (such as absorption and intellect), even though those facets are not correlated with each other [32]. Central facets therefore help explain patterns of covariance within a domain (e.g. people who are easily absorbed into their surroundings and those who are intellectual both tend to have vivid imaginations). Although there is general agreement on the gross features of each domain’s core, different trait measures are often anchored around different core facets (for a brief review, see [10]). The abundance of personality measures in the ESCS provides a unique opportunity to identify a transtheoretical core to each Big Five domain by triangulating across personality facets from many inventories.

In contrast, some facets may be peripherally associated with a domain, as evidenced by weak correlations with the domain’s other facets despite conceptual connections to the domain as a whole. For example, traditionalism may be a peripheral facet of conscientiousness that is only moderately correlated with other conscientiousness facets [33]. A clear-cut identification of peripheral facets is difficult because metrics for the boundaries between peripheral facets and facets beyond a domain remain unclear. Moreover, because different instruments measure different facets [10,34], researchers interested in comprehensively identifying domain peripheries must simultaneously examine facets from many different instruments.

Nevertheless, charting the periphery of trait domains can provide important insights into personality structure and potentially improve prediction in applied settings. Some research suggests that facet level scales are better predictors of certain outcomes than the Big Five domains ([22, 33], but see [35]). This would be particularly likely when facet scales contain specific outcome-related variance that is averaged-out when computing broader domain scores. As peripheral facets are less strongly associated with the core of a domain, they may be especially likely to contain unique variance not shared with the domain scale. Identifying the peripheral facets of a domain thus allows researchers to measure combinations of facets that contain unique variance, potentially improving the predictive power of trait models.

### Targeted understanding of particular facets or measures

Just as geneticists can use atlases to understand how different genes relate to one another, personality researchers could use a trait atlas to better understand a particular measure by examining its associations with other measures and its positioning within a domain. For example, a researcher interested in impulsivity could consult a facet atlas to investigate 1) the correlations between various impulsivity scales, 2) the extent to which impulsivity scales reflect blended content of multiple Big Five domains, and 3) whether these impulsivity scales fall in the core or periphery of each Big Five domain [36]. Together, this information may clarify cases of jingle and jangle, synthesize past research on impulsivity, and help researchers select impulsivity scales that are tuned for testing their specific hypotheses.

A facet atlas also allows researchers to test hypotheses concerning the structure of particular measures. The content of a personality measure reflects the joint effect of its creator’s beliefs about personality structure and evidence regarding those beliefs [17]. For example, the Big Five Aspects Scales (BFAS) were designed to measure two maximally distinct aspects within each of the Big Five domains [12]. In essence, the BFAS presents the hypothesis that variation within each Big Five domain can be summarized by two empirically-identified facets. It follows that 1) each scale should parsimoniously cover much of the domain’s space, 2) the two scales within a domain should be maximally distinctive from one another, and 3) each scale should be associated only with the domain it is intended to measure. The ESCS affords a relatively theory-neutral environment to test these structural theories because it contains scales from many personality measures.

### Examining facets using a network approach

In this study, we created a facet atlas by estimating and visualizing a series of network graphs. As the application of network modeling to the study of personality is relatively new, we offer a brief overview of this approach (for a more thorough review, see [37]). Network graphs visualize the connective patterns (e.g., correlations) among a set of variables. In personality networks, variables represented by circles (called nodes) are connected by lines (edges) that vary in width depending on the strength of correlation between the two variables. The nodes and edges are plotted in a two-dimensional space, such that nodes with similar patterns of correlation are plotted near one another, and nodes with dissimilar patterns of correlation are plotted farther apart [38]. Furthermore, nodes that correlate strongly with many other nodes are placed at the center of a network, whereas nodes with weaker correlations are placed nearer the periphery. Finally, centrality indices are computed that summarize each node’s position in the network.

Although cross-sectional personality network graphs and correlation matrices are based on the same information, network graphs present complex correlation patterns spatially, highlighting relevant information and summarizing each variable’s correlations with all other variables in the matrix. For instance, the core of a personality domain, which can be represented as the facets that are most strongly correlated overall with the other facets in the network, is visualized by placing strongly connected facets at the center of a network. The core or peripheral placement of a facet is thus much more easily identifiable in a network graph than in a large correlation matrix (which, in this study, would require 30 sheets of paper to display).

Network graphs also provide a complementary approach to factor analyses. Factor analysis allows researchers to distill a large covariance matrix into a smaller factor structure that captures the main dimensions of variability among items, such as the Big Five. Conversely, network graphs retain focus on the lower-level variables (in this case facets). As such, network visualizations depict individual variable-to-variable correlations, which are not the focus of factor analytic results. In sum, the workhorse of personality is the correlation matrix, and network models are a useful tool that facilitate the understanding of large, complex correlation matrices and complement factor analyses.

### The present study

In the present study, we used a network approach to examine patterns of correlation between 268 facets measured in 13 personality questionnaires that were administered to the ESCS. We estimated a network for each Big Five domain according to the results of an exploratory factor analysis of 268 facet scales. We visualized these networks to create a facet atlas, presented in this manuscript and as an online shiny app (https://tedschwaba.shinyapps.io/appdata/). We then explored how this atlas can be used to investigate blended personality facets as well as the core and periphery of each Big Five domain. Finally, we showcase how this facet atlas can be used to better understand particular constructs by examining facets measuring impulsivity, and we illustrate how it can test structural hypotheses contained within measures by examining the Big Five Aspect Scales.

## Methods

### Participants

ESCS participants were recruited through a mailed invitation in 1993 and completed a variety of personality questionnaires sent in separate mailers beginning in 1993 (N = 1,134), with 88% retention over the following 10 years. In 1993, participants ranged in age from 18–85 (M = 49.67, SD = 13.08), and 34.7% of participants were ages 40–49. The sample was composed of 57% women, 98.4% European-Americans, and 59% of participants had at least a college degree. The ethnic and geographical homogeneity of the ESCS limit the generalizability of results, as we note in the discussion section. The number of participants varies by scale, as questionnaires were over a period of 2 decades. The items in some scales were administered over a period of multiple years, leading some facet scales within the same questionnaire to have different sample sizes.

### Measures

In this study, we included omnibus scales with either explicit hierarchical structure, hierarchical structure validated in other studies, or that measure many traits, such that each trait would approximate the scope of a facet. We present these measures in Table 1. Information for each facet scale, including N, alpha, and Big Five factor loadings, is available online at https://osf.io/f47xu/, and a correlation matrix of all facets is available at https://osf.io/w682t/.

**Table 1:** Measures in the present study.

Scale	Reference	Year(s)	N	N items	N facets
Abridged Big Five Circumplex (AB5C)	[9,13]	1994–1996	795–917	485	45
Big Five Aspects Scales (BFAS)	[12]	1994–1996	905–945	98^a^	10
Big Five Inventory (BFI)	[39]	1998	703	35	10
Behavioral Inhibition System/Behavioral Approach System Scales (BIS/BAS)	[40]	2003	734	20	4
California Personality Inventory (CPI)	[14]	1994	792	462	20
HEXACO Personality Inventory	[8]	2003	737	192	24
Hogan Personality Inventory (HPI)	[41]	1997	739–742	193	35^b^
Jackson Personality Inventory-Revised (JPI-R)	[42]	1999	712	300	15
Multidimensional Personality Questionnaire (MPQ)	[43]	1999	733	276	11
NEO Personality Inventory-Revised (NEO-PI-R)	[44]	1994	857	240	30
Six Factor Personality Questionnaire (6FPQ)	[45]	1999	714	108	18
Sixteen Personality Factor Questionnaire, Fifth Edition (16PF)	[46]	1996	680	185	16
Temperament and Character Inventory	[47]	1997	727	295	30

^a^ = The BFAS scale includes 100 items total, 2 of which were not measured in the ESCS.

^b^ = In this study, we omitted nine HPI facets (science ability, intellectual games, education, math ability, good memory, reading, self-focus, impression management, appearance) that measured ability, as these scales did not correlate highly with other facet scales.

### Analytic strategy

Analyses were conducted in R [48] using the packages psych [49] and qgraph [50]. The raw ESCS data are publicly available at https://dataverse.harvard.edu/dataverse/ESCS-Data, and the cleaned data and R scripts used in this study are available at https://osf.io/cjz8e.

Before visualizing this facet atlas, we first organized all 268 ESCS facets into the Big Five domains using exploratory factor analysis with oblimin rotation. All facets with factor loadings greater than |.25| were included in that domain. This threshold was chosen after reviewing the results of the factor analysis because it offered the best balance between our desires to include some facets in multiple domains and exclude facets with minimal conceptual and empirical association to a given domain. Two facets, HPI Not Autonomous and HPI Not Spontaneous, did not load .25 on any domain and thus were not included in any network. This .25 factor loading threshold can be changed in the online app.

For each Big Five domain, we then estimated a full (rather than partial) correlation matrix, using pairwise deletion, which described associations among facets. Although psychological network research often uses partial correlation methods [51], we estimated full correlation matrices for each network. A partial correlation estimates the association between a predictor and outcome while controlling for all other predictor-outcome associations in the network. In the present case, many scales in each network measured the same, or highly similar constructs (e.g. there were multiple sociability scales in the extraversion network) As such, the partial correlation between CPI *sociability* and HPI *likes parties* would be estimated while controlling for HEXACO *sociability*, removing important sociability-related variance and rendering results uninterpretable (see [52]). Controlling for this variance also removes meaningful patterns of correlation that arise from common latent factors (i.e. the Big Five, [53]) and makes the overall network structure less stable and replicable [54]. Thus, for this study, bivariate correlations were more appropriate than partial correlations.

We also corrected all associations for measurement error. Measurement error deflates correlations measured with lower reliability and is partly a function of instrument length, such that shorter instruments are typically less reliable [55]. Because the facet scales in this study varied in length from 1 to 46 items, it was especially important to correct for measurement error due to scale length. As we lacked item-level information for some scales, we applied a rough correction for unreliability using Cronbach’s alpha [56]; see http://ipip.ori.org/newMultipleconstructs.htm for a list of scale alphas calculated using item-level ESCS data). This correction for unreliability can be toggled in the online app. Because some ESCS scales were administered years apart from one another, we note that some correlations may remain somewhat attenuated (see [57]).

Finally, we estimated the most central and peripheral facets for each Big Five domain by calculating each facet’s network strength centrality. Strength is the sum of the absolute values of all correlations that each facet has with all other facets in a network [58]. In this study we calculate strength as the average of all correlations so that this metric is comparable across Big Five domains and with other networks. Facets with higher strength are relatively strongly associated with the other facets in a network, so we consider them to be core. This approach to defining the core of a network is conceptually similar to previous research that has argued that core facets have the highest loadings on a latent domain factor (e.g., [59]), and we examine if this is empirically true, as well. The converse logic applies to peripheral facets. Despite substantial factor loadings on the overall Big Five domain, peripheral facets have low network strength and are weakly associated with the other facets in a domain.

Past research has highlighted the importance of estimating centrality index reliability [37]; although this is less of an issue when estimating networks from full correlations [54]. To construct 95% confidence intervals for each facet’s strength, we adapted code from the bootnet package [37] and simulated 1,000 bootstrapped iterations of each network. This code is available at https://osf.io/9j3pm/.

## Results

### Network visualization and shiny app

**Fig 1:**
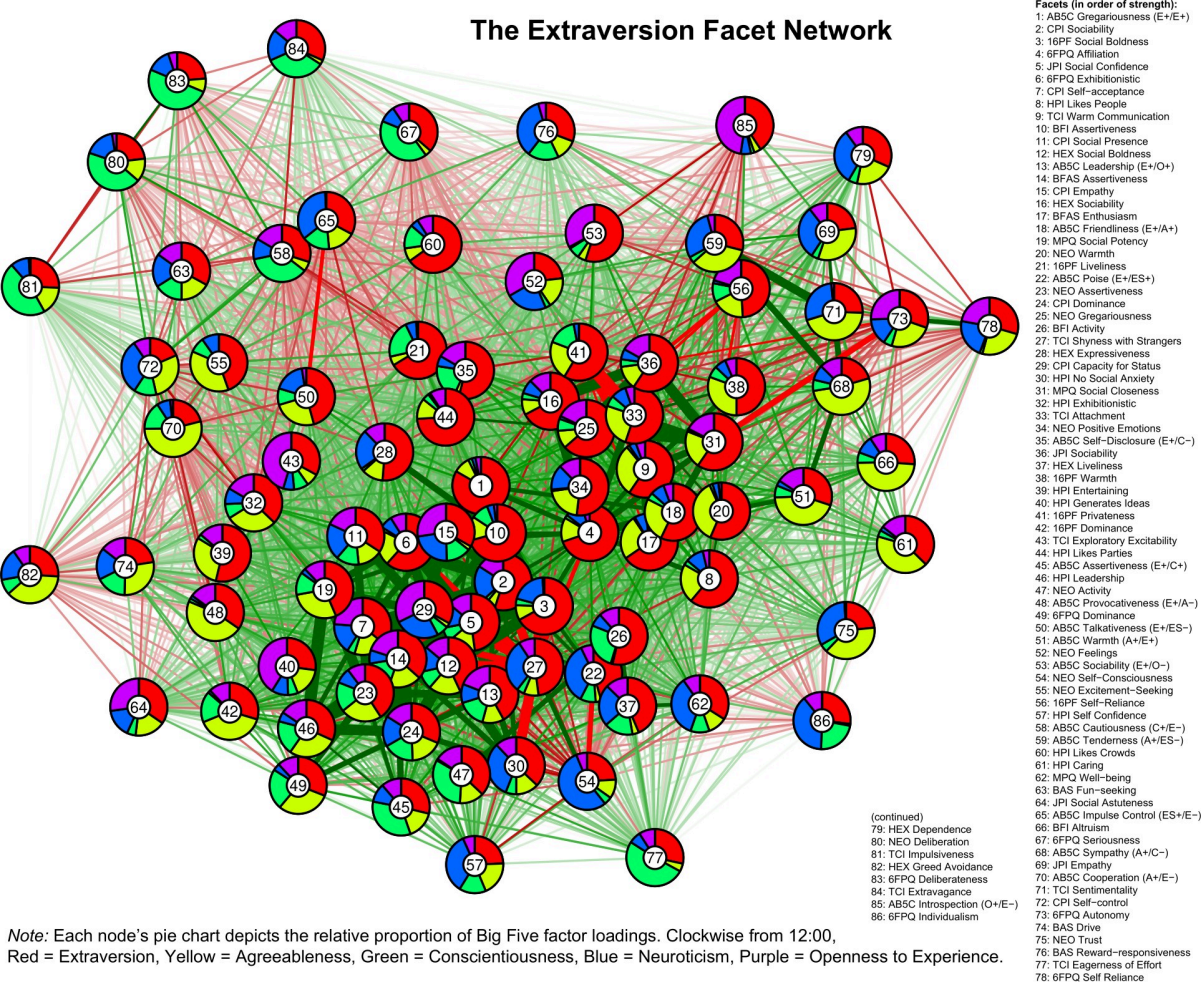
Extraversion facet network.

**Fig 2:**
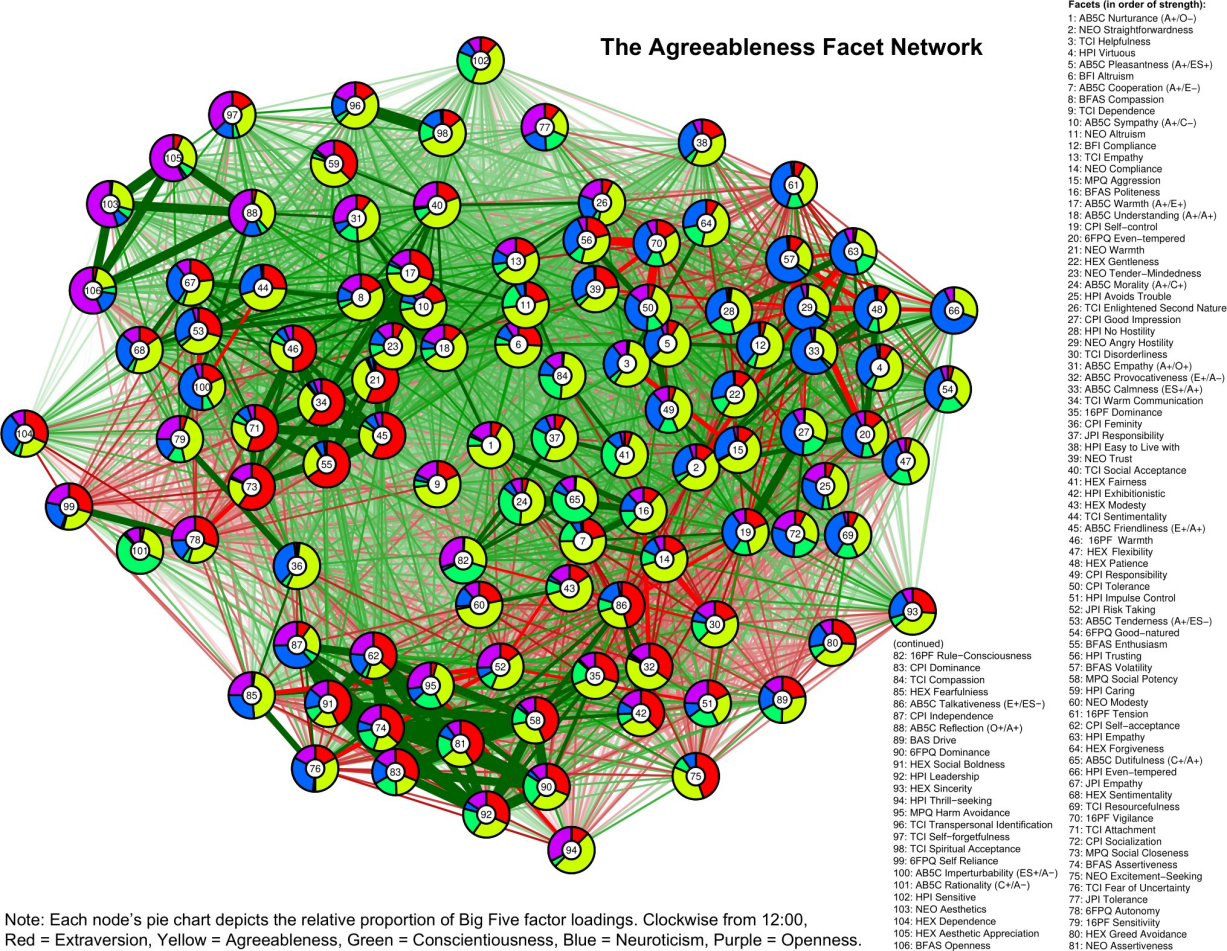
Agreeableness facet network.

**Fig 3:**
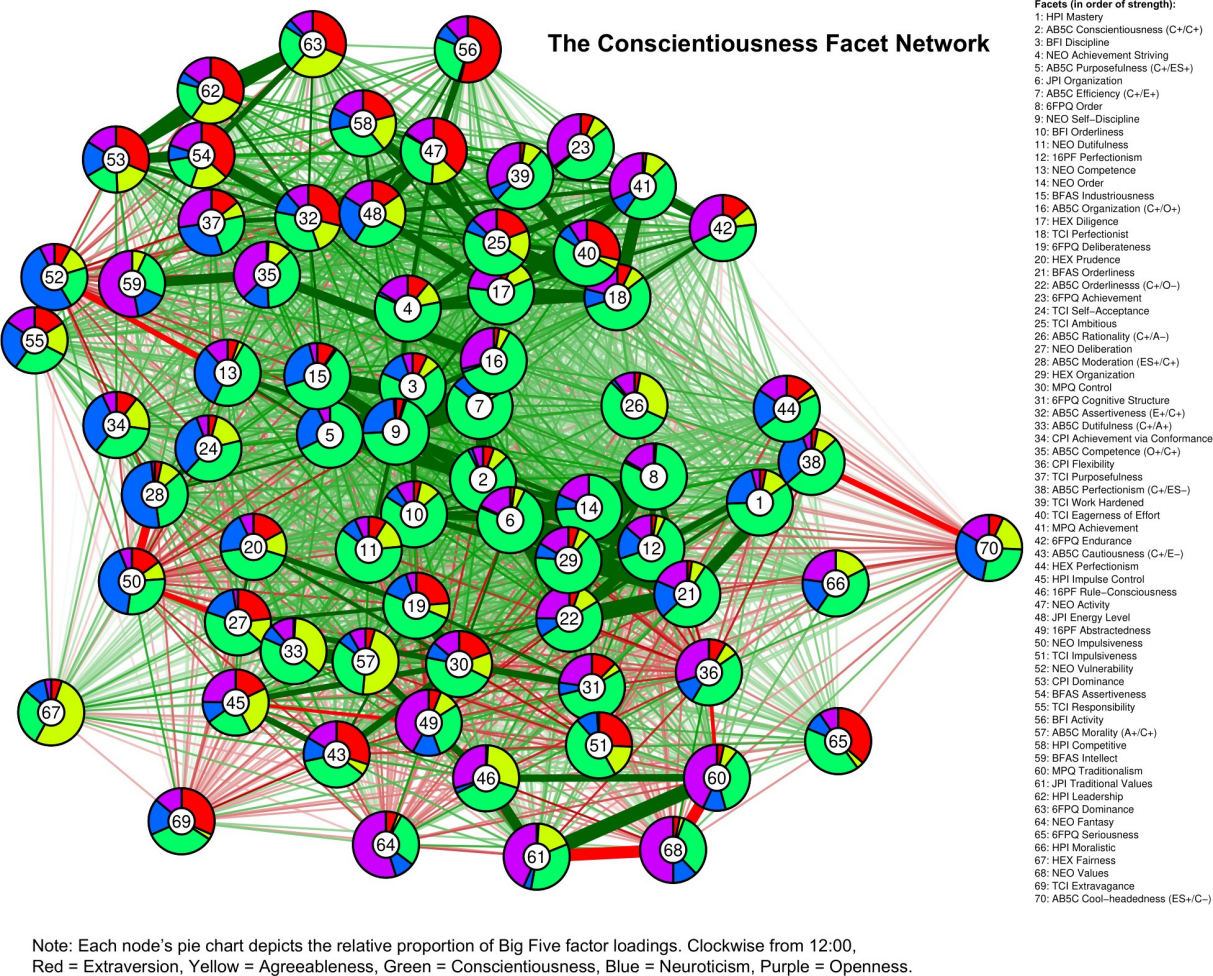
Conscientiousness facet network.

**Fig 4:**
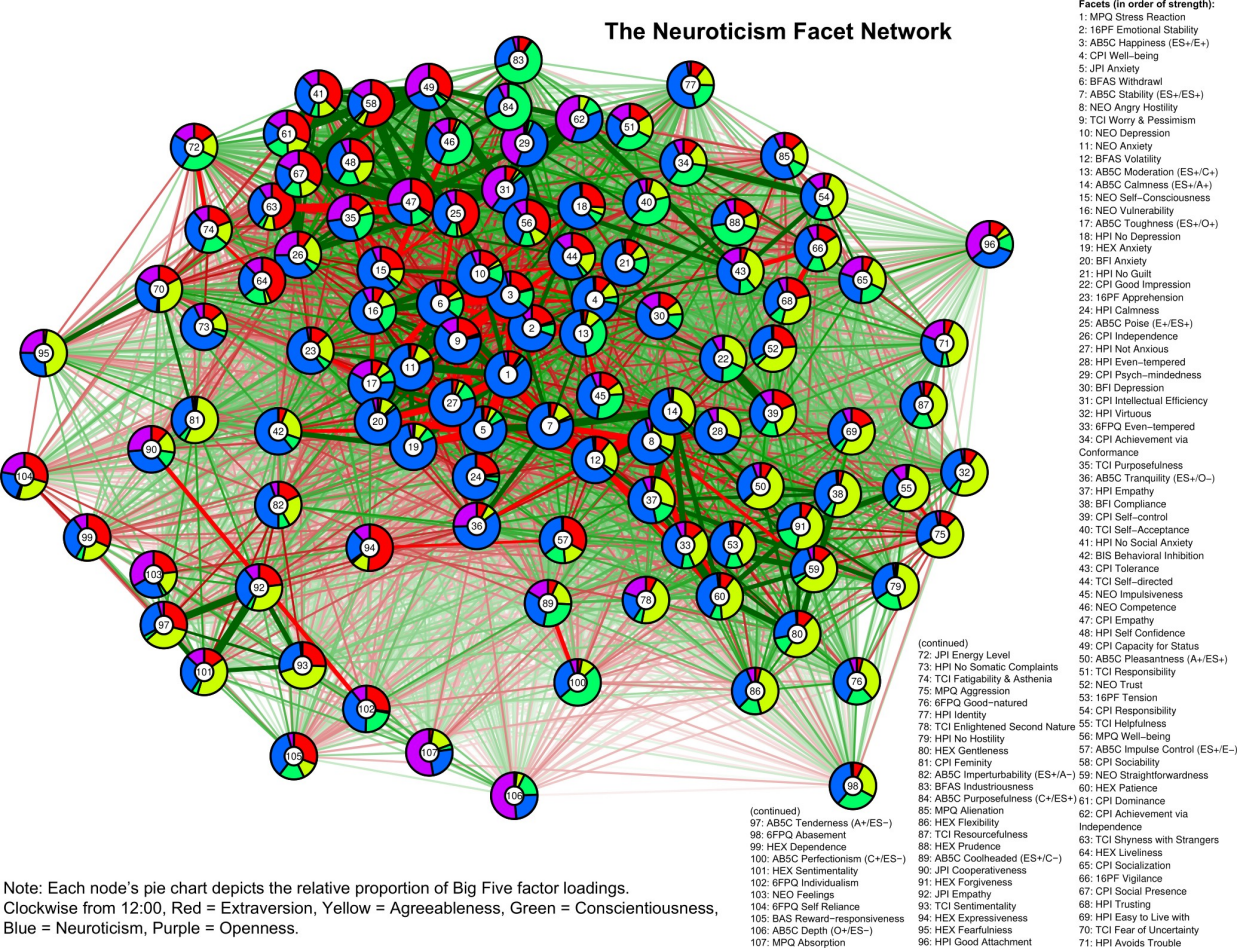
Neuroticism facet network.

**Fig 5:**
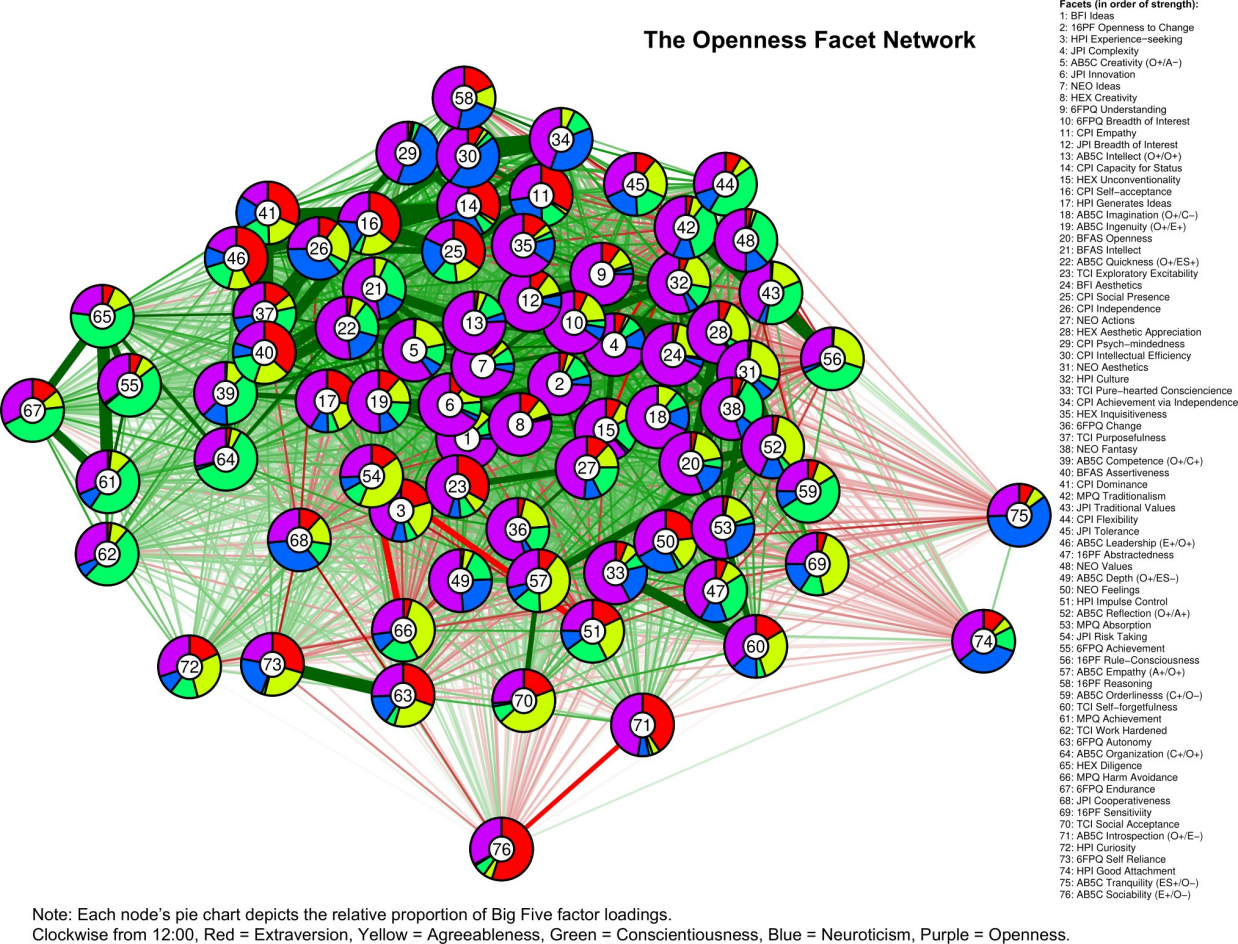
Openness facet network.

In Figs 1–5, we present a facet atlas, which displays the intercorrelations between the facets in each Big Five domain in rich network-based visualizations. For more information, we offer a point-and-click online app, hosted at https://tedschwaba.shinyapps.io/appdata/. In this app, users can explore a full network of all 268 facets, adjust the threshold for blended facets, and toggle the correction for measurement error. The app also displays complete descriptive information for all facet scales and allows users to visualize networks in terms of strength centrality. In the following sections, we describe how this facet atlas can be used to address major questions about the lower-level structure of personality outlined in the introduction.

### Blended facets

Results of the factor analysis suggested that the majority of facets (157 of 268, or 58.5%) contained a blended loading pattern with multiple factor loadings greater than |.25| (excluding the 40 AB5C facets designed to blended, 55.3% of facets were blended). Furthermore, 18 facets displayed blends with three domains, two facets displayed blends with four domains (BFAS *assertiveness* and J6F *self-reliance*) and one facet displayed blends with all five domains (CPI *dominance*). Blends with agreeableness (88 blends) were most common, followed by neuroticism (81), extraversion (76), openness (52) and conscientiousness (48). We summarize these patterns in Table 2 and visualize each facet’s blendedness in Figs 1–5.

**Table 2:** Summary of blended facet scales.

Blend	Number of scales	Example facets
E, A	22	NEO-PI-R Warmth, HEXACO Social Boldness
E, C	10	AB5C Cautiousness, NEO-PI-R Activity
E, N	12	TCI Shyness with Strangers, MPQ Well-Being
E, O	5	AB5C Introspection, TCI Exploratory Excitability
A, C	4	HEXACO Fairness, AB5C Rationality
A, N	35	BFAS Volatility, BFI Compliance
A, O	11	16PFQ Sensitivity, JPI-R Risk Taking
C, N	13	NEO-PI-R Impulsiveness, BFAS Orderliness
C, O	15	MPQ Achievement, CPI Flexibility
N, O	8	CPI Psychological-Mindedness, AB5C Tranquility
E, A, C	2	J6F Dominance, HPI Leadership
E, A, N	6	NEO-PI-R Trust, HEXACO dependency
E, A, O	2	CPI Self-Acceptance, J6F Autonomy
E, N, O	4	CPI Capacity for Status, NEO Feelings
A, C, O	2	16PFQ Rule-Consciousness, HPI Impulse Control
A, N, O	1	CPI Independence
C, N, O	1	TCI Purposefulness

Blended facets = factor loadings > |.25| on multiple factors. E = Extraversion. A = Agreeableness. C = Conscientiousness. N = Neuroticism. O = Openness. NEO-PI-R = NEO-Personality Inventory-Revised. AB5C = Abridged Big Five Circumplex. TCI = Temperament and Character Inventory. MPQ = Multiphasic Personality Questionnaire. BFAS = Big Five Aspects Scales. BFI = Big Five Inventory. 16PFQ = 16 Personality Factors Questionnaire. JPI = Jackson Personality Inventory-Revised. J6F = Jackson Six Factor Questionnaire. HPI = Hogan Personality Inventory. CPI = California Personality Inventory.

Facets with similar patterns of blendedness tended to cluster together, meaning that they had similar patterns of correlation with other facets in the network. For example, the top of the extraversion network contained a group of facets that were blends of extraversion, agreeableness, and low neuroticism. Positive and contrasting blends generally repulsed each other within a network, illustrating circumplex-like constellations. For example, the bottom-left side of the openness network contained facets with a contrasting blend of high openness and low agreeableness (such as 6FPQ *autonomy*), but the upper-right of the openness network contained facets with a positive blend of high openness and high agreeableness (such as 16PF *sensitivity*).

### Core and peripheral facets

**Fig 6:**
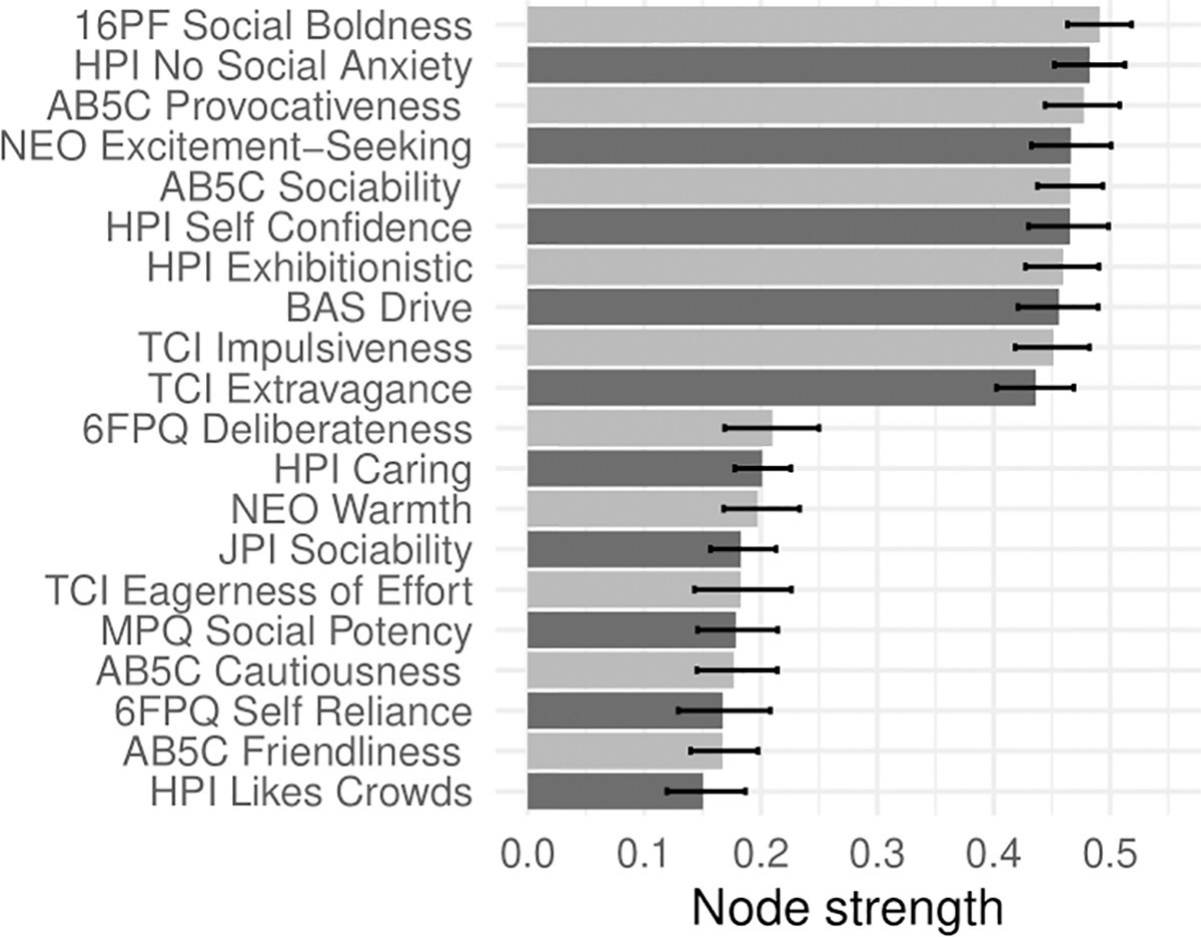
The most strongly and weakly connected facets in the extraversion network.

**Fig 7:**
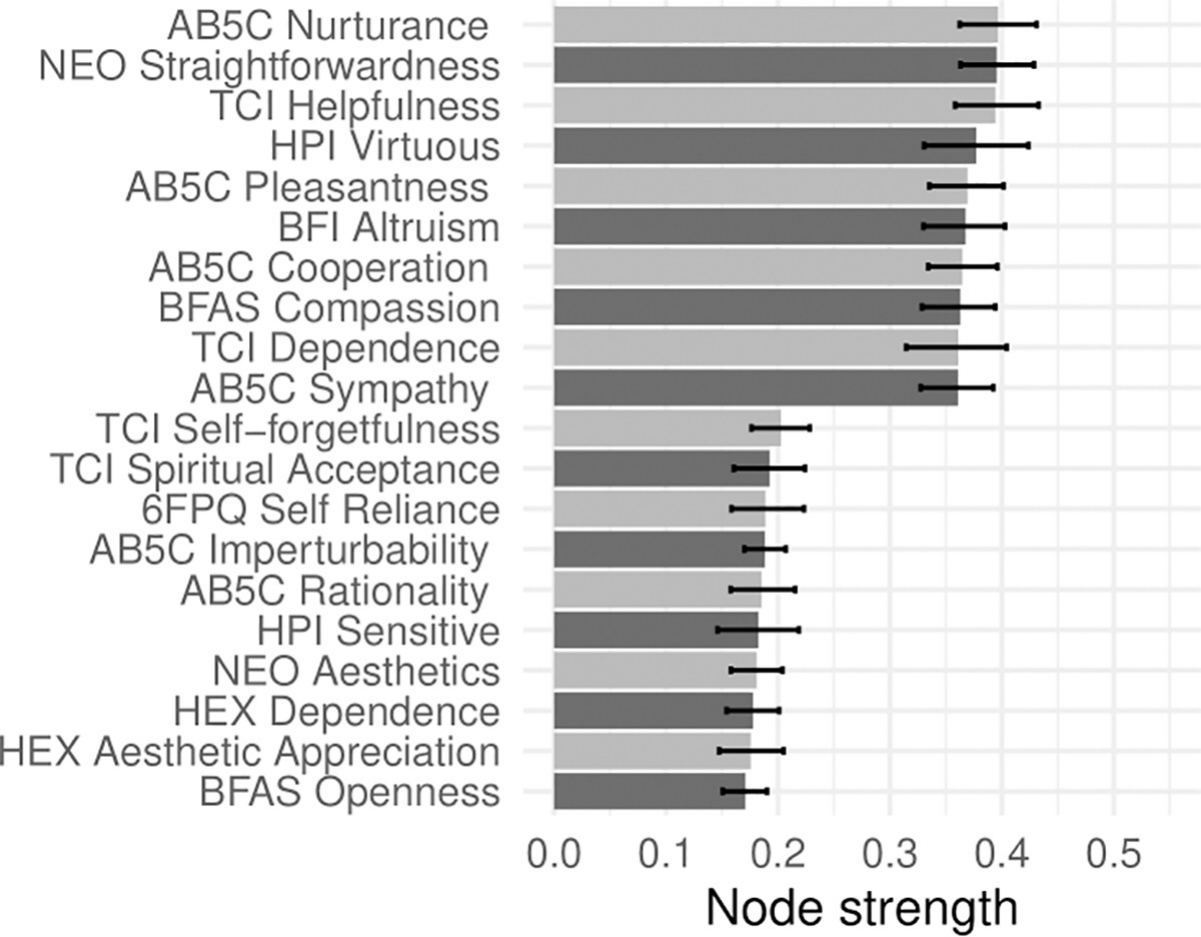
The most strongly and weakly connected facets in the agreeableness network.

**Fig 8:**
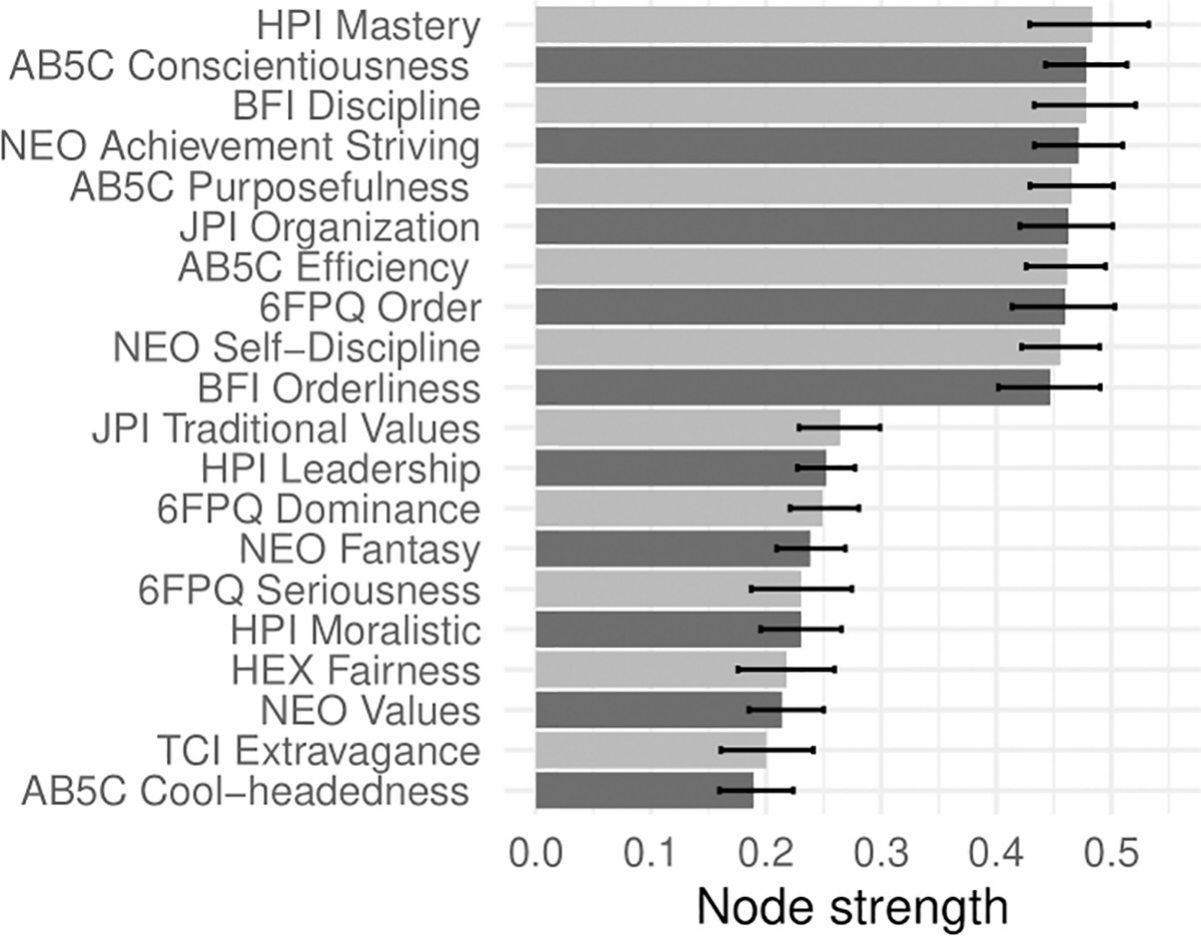
The most strongly and weakly connected facets in the conscientiousness network.

**Fig 9:**
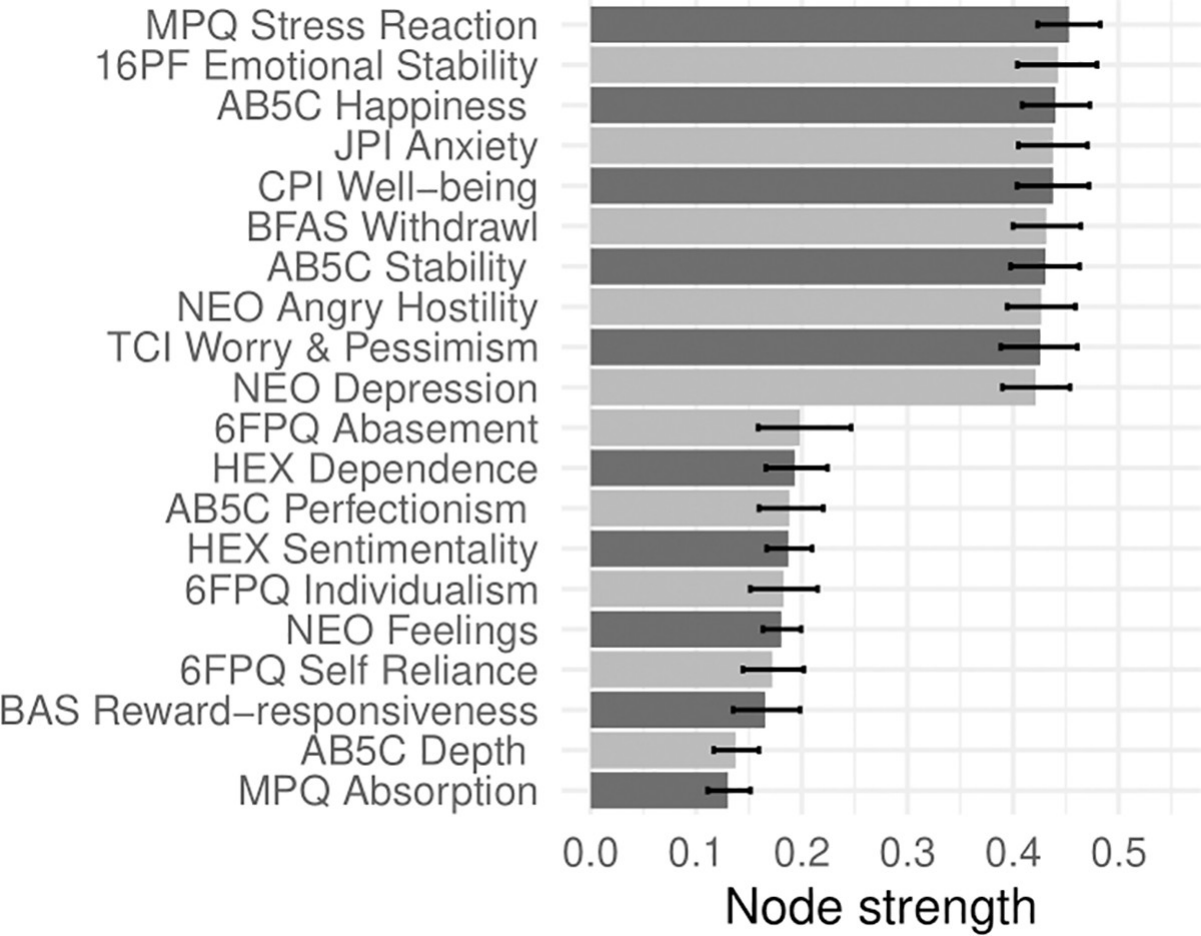
The most strongly and weakly connected facets in the neuroticism network.

**Fig 10:**
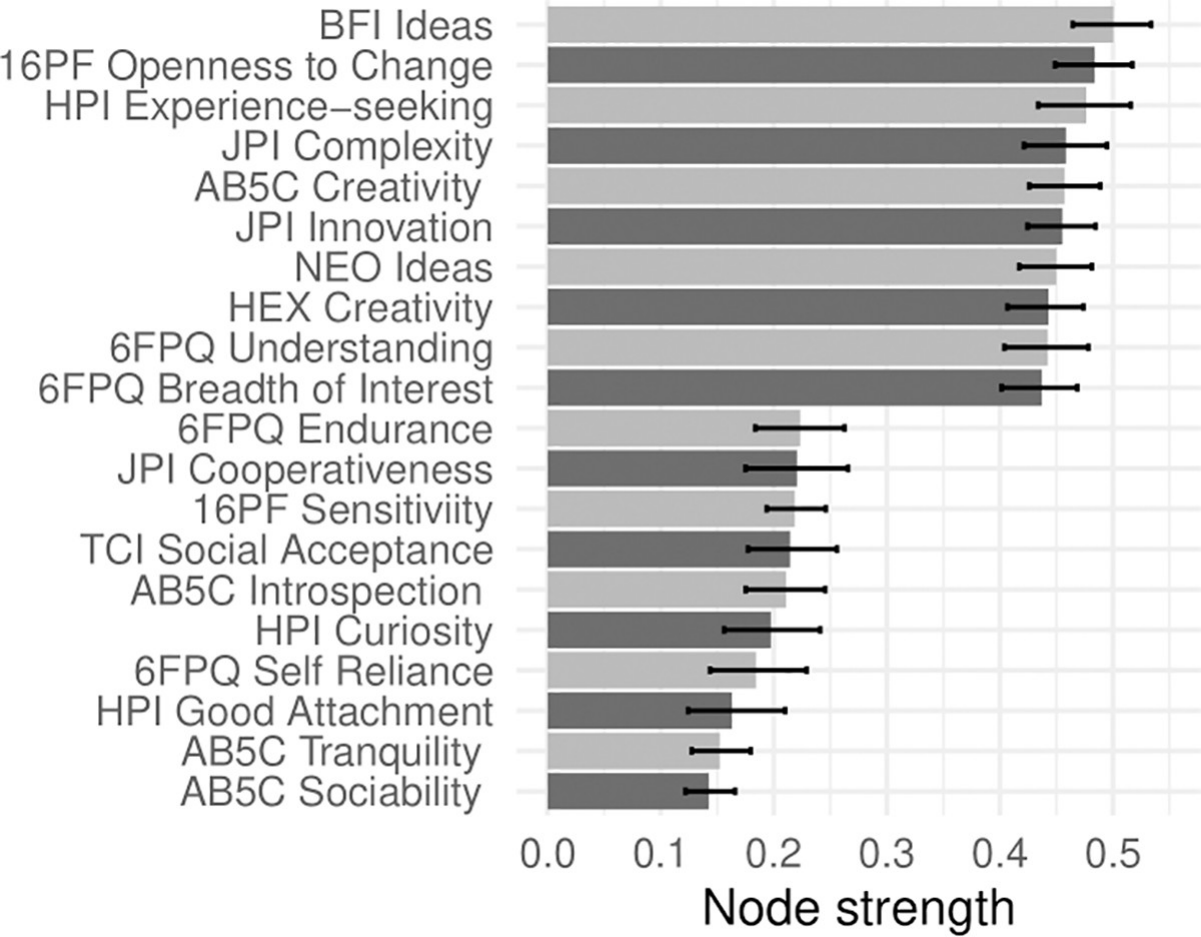
The most strongly and weakly connected facets in the openness network.

In Figs 1–5, nodes are labeled according to their strength, ranked from strongest to weakest. In Figs 6–10, we visualize network strength for each network’s 10 most core and 10 most peripheral facets. Strength estimates for all facets in each network are available at https://osf.io/cjz8e. Strength centrality estimates were highly correlated with the absolute value of factor loadings on that domain. Correlations were .82 for extraversion, .76 for agreeableness, .86 for conscientiousness, .79 for neuroticism, and .71 for openness

As can be seen in these figures, there were large differences in strength between the most peripheral facets and the most core facets. However, within each network’s core, no single scale or construct stood out as being most central. For example, within the set of the strongest agreeableness facets were scales measuring interpersonal warmth, altruism, and compassion, three related but distinct constructs. Possible exceptions were the neuroticism network, with core facets representing emotional reactivity and anxiety [12] and the extraversion network, with core facets primarily representing sociability [59].

The periphery of each domain’s network was also heterogenous in content and often contained blended facets. For example, the periphery of the conscientiousness network was composed of facets measuring diverse constructs including values (blended with openness), activity (blended with extraversion), and leadership (blended with extraversion and agreeableness). Overall, few facets appeared to be in conceptually unrelated networks where they did not belong, supporting our decision to use a factor loading of >|.25| for domain inclusion.

### Impulsivity in this facet atlas

This facet atlas can be used as a practical resource to understand a single measure or construct in more depth. We illustrate this by examining impulsivity. In the ESCS, four facet scales are labeled as some variant of impulsivity: NEO *impulsiveness*, TCI *impulsiveness*, AB5C *impulse control*, and HPI *impulse control*. These four scales had intercorrelations that ranged from .22 to .44 (Table 3), indicating that these scales measure a heterogenous set of constructs [36]. Accordingly, each impulsivity scale had a different blend of factor loadings: AB5C *impulse control* appeared in the neuroticism (factor loading = -.49) and extraversion (-.42) networks, NEO *impulsiveness* appeared in the neuroticism (.47) and conscientiousness (-.32) networks, HPI *impulse control* appeared in the agreeableness (.33), conscientiousness (.30) and openness (-.33) networks, and TCI *impulsiveness* appeared in the extraversion (.27) and conscientiousness (-.49) networks. This suggests that each of the scales besides AB5C *impulse control* measures conscientiousness-related features, and AB5C *impulse control* and NEO *impulsiveness* measure clinically relevant neuroticism-related features.

**Table 3:** Correlations between measures of impulsivity.

	1	2	3	4
1) AB5C Impulse Control		.43	-.65	-.34
2) HPI Impulse Control	.32		-.48	-.48
3) NEO PI-R Impulsiveness	-.44	-.32		.33
4) TCI Impulsiveness	-.26	-.35	.22	

N ranges from 727 to 900 across measures. All correlations are significant at *p* < .001. Correlations above the diagonal are corrected for measurement error using alpha. AB5C = Abridged Big Five circumplex. HPI = Hogan Personality Inventory. NEO PI-R = NEO Personality Inventory–Revised. TCI = Temperament and Character Inventory.

We turned to this facet atlas to better understand how these impulsivity scales relate to conscientiousness. In the conscientiousness network, HPI *impulse control* (strength = 22.15, 95% CI [19.77, 24.41]), NEO *impulsiveness* (strength = 21.34, 95% CI [18.49, 24.32]) and TCI *impulsiveness* (strength = 21.22, 95% CI [18.69, 23.66]) occupied similar, peripheral network positions, ranked 45^th^, 50^th^, and 51^st^ in network strength out of 70 facets, respectively. Researchers interested in predicting conscientiousness-related outcomes may therefore benefit from measuring impulsiveness using either of these three scales, as they may contribute additional predictive variance compared to core conscientiousness scales. This facet atlas also provides insight into the scales’ content. HPI *impulse control* was located near conscientiousness facets that measure deliberation, dutifulness, and cautiousness, suggesting this scale emphasizes the behavioral constraint components of impulsivity [36]. NEO *impulsiveness* was located near facets that measure moderation and prudence, suggesting that this scale emphasizes sensation-seeking components of impulsivity [36]. Finally, TCI *impulsiveness* was located near facets that measure flexibility and traditionalism, suggesting that this scale emphasizes components of impulsivity broadly related to rule-following and norm adherence. These differences indicate a jingle issue, where three distinct constructs are being operationalized as impulsiveness. Researchers interested in studying impulsiveness may benefit from considering these components separately or in tandem [60], and from paying close attention to the scales used in past research on impulsiveness.

The two impulsiveness scales with substantial loadings on neuroticism, AB5C *impulse control* (strength = 32.23, 95% CI [28.49, 36.13]) and NEO *impulsiveness* (strength = 34.49, 95% CI [30.05, 38.61]) occupied an intermediate space between the core and periphery of neuroticism, ranked 57^th^ and 42^nd^ in strength out of 107 facets. This indicates that impulsiveness as measured by these scales is moderately related to other neuroticism facets. AB5C *impulse control* and NEO *impulsiveness* also occupied similar network positions in the neuroticism network, demonstrating that these scales measure similar neuroticism-related content, despite their moderate correlation (*r* = .44). Nearby neuroticism facets measure constructs such as volatility, angry hostility, and (low) cool-headedness, indicating that these two scales include components of both general affective reactivity and quickness to anger [61], which appears to be omitted from the TCI *impulsiveness* scale and HPI *impulse control* scale.

### The BFAS in this facet atlas

A facet atlas can be used to evaluate the implicit theories of trait structure inherent in most personality instruments. We illustrate this feature by examining the network placement of facets measured in the Big Five Aspects Scales (BFAS; [12]). We note that the BFAS was created by factor-analyzing the AB5C and NEO-PI-R scales in the ESCS dataset. However, the present analyses include correlational patterns with 191 additional facet scales, revealing new information about how the BFAS scales measure personality traits.

Because the BFAS was created by factor analysis, the two aspect scales for each of the Big Five should both parsimoniously cover much of the domain’s space. This can be evaluated by examining whether each domain’s aspect scales fall within its core. This was the case for each of the five domains (see Figs 1–6). The most peripheral aspect scale was *orderliness* (ranked 21st of all 70 facet scales in the conscientiousness network). Each domain’s aspect scales did not differ significantly in strength from one another, suggesting that both scales were equally core (i.e. no aspect was “more important” than the other).

A consequence of the specific factor-analytic procedure used to derive the BFAS is that, because the two aspect scales in each domain were rotated to be orthogonal, they should each measure distinctly different content. This can be evaluated by examining how far apart the two aspects are within each domain’s network, as scales that have similar patterns of correlations with other facets will occupy network positions close to one another. Results indicated that the two BFAS aspect scales in each domain were indeed placed relatively far apart from one another (while still remaining in the domain’s core), supporting the idea that these scales cover a relatively broad content area within each domain.

We also explored the extent to which BFAS aspect scales contained blended content from multiple Big Five domains. Results indicated that six out of 10 aspect scales were exclusively associated with a single domain, whereas four scales (*assertiveness*, *industriousness*, *intellect*, and *openness*) were associated with multiple domains at a cutoff of |.25|. Most notably, BFAS *assertiveness* was a blend of four Big Five domains: extraversion, (low) agreeableness, openness, and conscientiousness. This suggests that, although each aspect scale was derived using items that measured a single domain, some are not factor-pure markers of a single domain [62].

## Discussion

In this study, we created a facet atlas to organize and summarize the current state of facet-level personality trait measurement. To illustrate the utility of this atlas, we examined the prevalence of blended facets, identified core and peripheral facets for each of the Big Five, and explored how researchers can use this facet atlas to better understand particular constructs and measures. In what follows, we discuss implications of this research for applied personality assessment and our conceptual understanding of personality trait structure.

### The prevalence of blended facets

Most scales (59%) contained a blend of multiple Big Five domains. Although we are certainly not the first to point out the lack of simple structure in the personality hierarchy (see [11,15,30]), basic and applied personality researchers seem reluctant to incorporate this complex, blended reality into personality assessment and theory, as evidenced by the simple structure implied in most non-circumplicial personality measures (e.g. [8,10,12]). More fully acknowledging the prevalence of blended content in facet scales can increase commensurability between measures and improve structural theories.

Results also indicated that three specific domain combinations were commonly represented through blends. One of these, the blend between agreeableness and extraversion, reflects the well-known interpersonal circumplex [23]. Two other blends, between agreeableness and neuroticism and between conscientiousness and openness, were also common but have been given less empirical attention. Blends of agreeableness and neuroticism may form a circumplex measuring interpersonal affective tendencies [31]. Conceptualizing such a circumplex may be useful in the diagnosis and treatment of interpersonal problems. The blend between conscientiousness and openness may form a circumplex based around a person’s value system (high C high O = ego development, High C low O = rigidity, low C high O = unconventionality, low O low C = disengagement). This circumplex may be useful for understanding humanistic aspects of personality and may aid in synthesizing Big Five personality research with that on ego development (e.g., [63]).

Two particular types of facet blends, between extraversion and openness, and between agreeableness and conscientiousness, were uncommon in this facet atlas. This finding was relatively surprising, as these two pairs of domains are typically intercorrelated [4, 13]. The paucity of blends between facets of extraversion and openness does not appear to reflect empty space, as we did identify a few positive blends (TCI exploratory excitability and AB5C leadership) and a few contrasting blends (AB5C introspection and sociability). Also, past circumplicial research has also found that facets measuring ingenuity, creativity, and bold leadership measure a blend of high openness and high extraversion [30,64]. Rather, it seems that scales measuring blends of extraversion and openness are just uncommon. Developing scales that explicitly measure a blend of these traits may be useful, as they form the metatrait of plasticity and have been theorized to function in tandem as part of the approach system [65]. In comparison, the lack of blended content between agreeableness and conscientiousness may reflect trait space that is necessarily sparser. We found one negative blend (AB5C rationality) and three positive blends (AB5C dutifulness, AB5C morality, and HEXACO fairness), and other research has similarly struggled to identify blended content between these two traits [65]. We note that each of these four facets connotes some sort of interpersonally-focused rule adherence, which is a common behavior in everyday life but does not seem to be well-encoded into the language of stable individual differences (as evidenced by the fact that we cannot identify a single-adjective term to describe this kind of behavior). Future research may wish to further delve into the personality space occupied by a blend of agreeableness and conscientiousness, and to develop scales that explicitly measure this content. Doing so could augur a more comprehensive approach to personality assessment.

### The cores of each domain

We identified the most core and peripheral facets in each Big Five domain by computing each facet’s strength within the respective network. A heterogenous set of facets characterized the five domain cores. For example, the core of conscientiousness contained facets measuring content including mastery, purposefulness, and organization. These findings identify cases of jingle and jangle in facet names. For example, AB5C *sociability* was located in the core of extraversion and JPI *sociability* was in the periphery, though they share the same name. This pattern of findings also highlights the inherent difficulty in identifying a single conceptual “core” for each of the broad Big Five domains. Rather, a domain’s core may be best understood as collection of facets, and the positioning of facets may be best done in relative terms (e.g. as “more core” or “more peripheral” than another facet.)

We also found that, within each domain, network centrality estimates were highly correlated with the absolute value of factor loadings in that domain (rs = .71-.86). The magnitude of this correlation, though smaller than the near-unity correlations between these two parameters when estimated in simulation studies [66], suggests that similar information is gleaned from both kinds of analyses (we note that the simulations estimated networks based on partial correlations, whereas we estimated networks based on full correlations). The major source of discrepancy between the two estimates likely comes from the fact that factor analysis summarize how facets are similar in terms of their associations with a single broader Big Five domain, whereas strength centrality estimates summarize all sources of similarity and difference between each pair of facets. For example, TCI social acceptance and AB5C empathy both load strongly on a latent agreeableness factor, but this association is made even stronger by virtue of a shared secondary loading on openness, which is solely captured in network strength estimates. Overall, this overlap between factor analytic and network analytic results suggests that the two methodologies share many features, especially when network analyses are based on cross-sectional correlations.

### The peripheries of each domain

Whereas much attention has been paid to identifying the cores of each of the Big Five domains, this study was one of the first to examine the peripheral facets of the five domains. Results indicated that each domain contained substantial peripheral content that was covered by few measures. Along with past research [34, 36], this suggests that most modern personality measures have idiosyncratic breadth in their content coverage. For example, the NEO-PI-R and HPI measure *trust*, a facet of agreeableness, but the other hierarchical personality measures in the ESCS do not. These differences in peripheral content coverage may account, in part, for the moderate correlations that have been reported for different measures of the same Big Five domain (e.g. as low as *r* = .66 in [10]). Future research that focuses on the periphery of the trait domains can help resolve differences in domain scores from different measures, clarify how different instruments are more or less effective at accounting for certain traits, and improve efforts to comprehensively assess personality.

### Limitations

The major limitations of this research involve the composition of the ESCS. The sample is ethnically homogenous; over 98% of participants are white, all are Americans, and most are middle-aged. As the structure of personality traits, especially at the facet level, does not generalize across cultures [67] or age groups [68], researchers should be cautious when generalizing this atlas to different groups of people. As a broader point, the ESCS has been heavily utilized in past examinations of personality structure (e.g. [10, 12, 30)]) because participants have completed such a wide variety of personality measures. The unfortunate side effect of this overreliance on the ESCS and samples with similar composition is that our research on personality structure often excludes broader nonwhite populations, even within the US. To rectify this, future work that collects data used to study personality structure using many facet scales must actively focus on sample diversity (such as [69]). We eagerly anticipate future, more representative atlases.

In addition, all of the instruments in this study were self-report questionnaires, and results may differ when using a different method. Additionally, some facet scales were measured with few items, and this brevity introduces measurement unreliability. We corrected for this using each scale’s alpha reliability, but this rough correction is relatively conservative and may not restore each correlation to its actual magnitude. As such, correlations between facets measured with brief scales may be somewhat attenuated.

## Conclusion

We created a facet atlas that organizes the current state of facet-level measurement by describing connections between different lower-order personality trait scales. A better understanding of facets can assist in individual case formulation [28], clarify trends in personality development [70], aid in the prediction of important life outcomes [71], and refine theories of personality structure [72]. Ideally, this atlas can serve as a reference guide to personality scholars of all stripes who wish to use facet scales in research and applied settings.

## References

[1] John OP, Naumann LP, Soto CJ. Paradigm shift to the integrative Big Five trait taxonomy: History, measurement, and conceptual issues In: Handbook of personality: Theory and research, 3rd ed New York, NY, US: The Guilford Press; 2008 p. 114–58.

[2] Eysenck HJ. Dimensions of personality. Oxford, England: Kegan Paul; 1947. 308 p. (Dimensions of personality).

[3] Digman JM. Personality structure: Emergence of the five-factor model. Annual Review of Psychology. 1990;41:417–40.

[4] Mõttus R, Kandler C, Bleidorn W, Riemann R, McCrae RR. Personality traits below facets: The consensual validity, longitudinal stability, heritability, and utility of personality nuances. Journal of Personality and Social Psychology. 2017;112(3):474–90. 10.1037/pspp0000100 27124378

[5] Markon KE, Krueger RF, Watson D. Delineating the Structure of Normal and Abnormal Personality: An Integrative Hierarchical Approach. Journal of Personality and Social Psychology. 2005;88(1):139–57. 10.1037/0022-3514.88.1.139 15631580 PMC2242353

[6] Wright AGC, Thomas KM, Hopwood CJ, Markon KE, Pincus AL, Krueger RF. The hierarchical structure of DSM-5 pathological personality traits. Journal of Abnormal Psychology. 2012;121(4):951–7. 10.1037/a0027669 22448740 PMC3389150

[7] Kotov R, Krueger RF, Watson D, Achenbach TM, Althoff RR, Bagby RM, et al The Hierarchical Taxonomy of Psychopathology (HiTOP): A dimensional alternative to traditional nosologies. Journal of Abnormal Psychology. 2017;126(4):454–77. 10.1037/abn0000258 28333488

[8] Lee K, Ashton MC. Psychometric Properties of the HEXACO Personality Inventory. Multivariate Behavioral Research. 2004 4;39(2):329–58. 10.1207/s15327906mbr3902_8 26804579

[9] Goldberg LR. A broad-bandwidth, public domain, personality inventory measuring the lower-level facets of several five-factor models In: Mervielde I, Deary IF, De Fruyt F, Ostendorf F, editors. Personality Psychology in Europe. Tilburg, The Netherlands: Tilburg University; 1999 p. 7–28.

[10] Soto CJ, John OP. The next Big Five Inventory (BFI-2): Developing and assessing a hierarchical model with 15 facets to enhance bandwidth, fidelity, and predictive power. Journal of Personality and Social Psychology. 2017;113(1):117–43. 10.1037/pspp0000096 27055049

[11] Ziegler M, Bäckström M. 50 Facets of a Trait– 50 Ways to Mess Up? European Journal of Psychological Assessment. 2016 4;32(2):105–10.

[12] DeYoung CG, Quilty LC, Peterson JB. Between facets and domains: 10 aspects of the Big Five. Journal of Personality and Social Psychology. 2007;93(5):880–96. 10.1037/0022-3514.93.5.880 17983306

[13] Hofstee WK, de Raad B, Goldberg LR. Integration of the Big Five and circumplex approaches to trait structure. Journal of Personality and Social Psychology. 1992;63(1):146–63. 10.1037//0022-3514.63.1.146 1494982

[14] Gough HG. CPI Manual: Third Edition Palo Alto, CA: Consulting Psychologists Press;

[15] Condon DM, Mroczek DK. Time to move beyond the big five? Eur J Pers. 2016;30(4):311–2. 29491551 PMC5826584

[16] Block J. Three tasks for personality psychology In: Developmental science and the holistic approach. Mahwah, NJ, US: Lawrence Erlbaum Associates Publishers; 2000 p. 155–64.

[17] Loevinger J. Objective Tests as Instruments of Psychological Theory. Psychol Rep. 1957 6 1;3(3):635–94.

[18] Bulik-Sullivan B, Finucane HK, Anttila V, Gusev A, Day FR, Loh P-R, et al An atlas of genetic correlations across human diseases and traits. Nature Genetics. 2015 11;47(11):1236–41. 10.1038/ng.3406 26414676 PMC4797329

[19] Hathaway SR, Meehl PE. An atlas for the clinical use of the MMPI. Oxford, England: U. Minnesota Press; 1951. 799 p. (An atlas for the clinical use of the MMPI).

[20] Goldberg LR, Saucier G. The Eugene-Springfield Community Sample: Information Available from the Research Participants [Internet]. ORI Technical Report; 2016. Available from: https://ipip.ori.org/ESCS_TechnicalReport_January2016.pdf

[21] Johnson JA, Ostendorf F. Clarification of the five-factor model with the Abridged Big Five Dimensional Circumplex. Journal of Personality and Social Psychology. 1993;65(3):563–76.

[22] Paunonen SV. Hierarchical organization of personality and prediction of behavior. Journal of Personality and Social Psychology. 1998;74(2):538–56.

[23] Wiggins JS. A psychological taxonomy of trait-descriptive terms: The interpersonal domain. Journal of Personality and Social Psychology. 1979;37(3):395–412.

[24] Leary T. Interpersonal diagnosis of personality: a functional theory and methodology for personality evaluation. New York: Wiley; 1980.

[25] McCrae RR, Costa PT. The structure of interpersonal traits: Wiggins’s circumplex and the five-factor model. Journal of Personality and Social Psychology. 1989;56(4):586–95. 10.1037//0022-3514.56.4.586 2709308

[26] Markey P, Lowmaster S, Eichler W. A real-time assessment of interpersonal complementarity. Personal Relationships. 2010;17(1):13–25.

[27] Sadler P, Ethier N, Gunn GR, Duong D, Woody E. Are we on the same wavelength? Interpersonal complementarity as shared cyclical patterns during interactions. Journal of Personality and Social Psychology. 2009;97(6):1005–20. 10.1037/a0016232 19968416

[28] Pincus AL, Cain NM. Interpersonal Psychotherapy In: Richard DCS, Huprich SK, editors. Clinical psychology: Assessment, treatment, and research. San Diego, CA: Academic Press; 2008 p. 213–45.

[29] Tracey TJ. An interpersonal stage model of the therapeutic process. Journal of Counseling Psychology. 1993;40(4):396–409.

[30] Woods SA, Anderson NR. Toward a periodic table of personality: Mapping personality scales between the five-factor model and the circumplex model. Journal of Applied Psychology. 2016 4;101(4):582–604. 10.1037/apl0000062 26653532

[31] Saucier G. Benchmarks: Integrating affective and interpersonal circles with the Big-Five personality factors. Journal of Personality and Social Psychology. 1992;62(6):1025–35.

[32] DeYoung CG, Grazioplene RG, Peterson JB. From madness to genius: The Openness/Intellect trait domain as a paradoxical simplex. Journal of Research in Personality. 2012 2 1;46(1):63–78.

[33] Roberts BW, Chernyshenko OS, Stark S, Goldberg LR. The structure of conscientiousness: An empirical investigation based on seven major personality questionnaires. Personnel Psychology. 2005 3;58(1):103–39.

[34] Christensen AP, Cotter KN, Silvia PJ. Reopening Openness to Experience: A Network Analysis of Four Openness to Experience Inventories. Journal of Personality Assessment. 2019 11 2;101(6):574–88. 10.1080/00223891.2018.1467428 29746176

[35] Grucza RA, Goldberg LR. The Comparative Validity of 11 Modern Personality Inventories: Predictions of Behavioral Acts, Informant Reports, and Clinical Indicators. Journal of Personality Assessment. 2007 11 1;89(2):167–87. 10.1080/00223890701468568 17764394

[36] Whiteside SP, Lynam DR. The Five Factor Model and impulsivity: using a structural model of personality to understand impulsivity. Personality and Individual Differences. 2001 3 1;30(4):669–89.

[37] Epskamp S, Borsboom D, Fried EI. Estimating psychological networks and their accuracy: A tutorial paper. Behav Res. 2018 2 1;50(1):195–212. 10.3758/s13428-017-0862-1 PMC5809547 28342071

[38] Fruchterman TMJ, Reingold EM. Graph drawing by force-directed placement. Softw: Pract Exper. 1991 11;21(11):1129–64.

[39] Soto CJ, John OP. Ten facet scales for the Big Five Inventory: Convergence with NEO PI-R facets, self-peer agreement, and discriminant validity. Journal of Research in Personality. 2009 2 1;43(1):84–90.

[40] Carver CS, White TL. Behavioral inhibition, behavioral activation, and affective responses to impending reward and punishment: The BIS/BAS Scales. Journal of Personality and Social Psychology. 1994;67(2):319–33.

[41] Hogan R, Hogan J. Hogan Personality Inventory Manual. 2nd ed Tulsa, OK: Hogan Assessment Systems; 1992.

[42] Jackson DN. Jackson Personality Inventory—Revised manual. Port Huron, MI: Sigma Assessment Systems; 1994.

[43] Tellegen A. Multidimensional Personality Questionnaire test booklet. 2nd ed Minneapolis, MN: University of Minnesota Press; 2003.

[44] McCrae RR, Costa PT Jr. Revised NEO personality inventory (NEO-PI-R) and NEO five-factor inventory (NEO-FFI) professional manual. Odessa, FL: Psychological Assessment Resources; 1992.

[45] Jackson DN, Paunonen SV, Tremblay PF. Six Factor Personality Questionnaire: Technical Manual. Port Huron, MI: Sigma Assessment Systems; 2000.

[46] Conn SR, Rieke ML. 16PF fifth edition technical manual. Institute for Personality & Ability Testing, Incorporated; 1994.

[47] Cloninger CR, Przybeck TR, Svrakic DM, Wetzel RD. The Temperament and Character Inventory (TCI): A guide to its development and use. 1994;

[48] R Core Team. R: A language and environment for statistical computing [Internet]. Vienna, Austria: R Foundation for Statistical Computing; 2018 Available from: https://www.R-project.org/

[49] Revelle WR. psych: Procedures for Personality and Psychological Research. 2017.

[50] Epskamp S, Cramer AOJ, Waldorp LJ, Schmittmann VD, Borsboom D. qgraph: Network Visualizations of Relationships in Psychometric Data. Journal of Statistical Software [Internet]. 2012;48(i04). Available from: https://ideas.repec.org/a/jss/jstsof/v048i04.html

[51] Bringmann LF, Eronen MI. Don’t blame the model: Reconsidering the network approach to psychopathology. Psychological Review. 2018 7;125(4):606–15. 10.1037/rev0000108 29952625

[52] Miller GA, Chapman JP. Misunderstanding analysis of covariance. Journal of Abnormal Psychology. 2001;110(1):40–8. 10.1037//0021-843x.110.1.40 11261398

[53] Molenaar PCM. Latent variable models are network models. Behavioral and Brain Sciences. 2010 6;33(2–3):166–166. 10.1017/S0140525X10000798 20584385

[54] Forbes MK, Wright AGC, Markon KE, Krueger RF. Evidence that psychopathology symptom networks have limited replicability. Journal of Abnormal Psychology. 2017;126(7):969–88. 10.1037/abn0000276 29106281 PMC5749927

[55] Revelle W, Condon DM. Reliability from α to ω: A tutorial. Psychological Assessment. 2019;31(12):1395–411. 10.1037/pas0000754 31380696

[56] Spearman C. The Proof and Measurement of Association between Two Things. The American Journal of Psychology. 1904 1;15(1):72–101. 3322052

[57] Anusic I, Schimmack U. Stability and change of personality traits, self-esteem, and well-being: Introducing the meta-analytic stability and change model of retest correlations. Journal of Personality and Social Psychology. 2016;110(5):766–81. 10.1037/pspp0000066 26619304

[58] Opsahl T, Agneessens F, Skvoretz J. Node centrality in weighted networks: Generalizing degree and shortest paths. Social Networks. 2010 7 1;32(3):245–51.

[59] Lucas RE, Diener E, Grob A, Suh EM, Shao L. Cross-cultural evidence for the fundamental features of extraversion. Journal of Personality and Social Psychology. 2000;79(3):452–68. 10.1037//0022-3514.79.3.452 10981846

[60] Nigg JT. Annual Research Review: On the relations among self-regulation, self-control, executive functioning, effortful control, cognitive control, impulsivity, risk-taking, and inhibition for developmental psychopathology. Journal of Child Psychology and Psychiatry. 2017;58(4):361–83. 10.1111/jcpp.12675 28035675 PMC5367959

[61] Maples J, Miller JD, Hoffman BJ, Johnson SL. A test of the empirical network surrounding affective instability and the degree to which it is independent from neuroticism. Personality Disorders: Theory, Research, and Treatment. 2014;5(3):268–77. 10.1037/per0000019 23834514

[62] DeYoung CG, Weisberg YJ, Quilty LC, Peterson JB. Unifying the Aspects of the Big Five, the Interpersonal Circumplex, and Trait Affiliation: Big Five and IPC. J Pers. 2013 10;81(5):465–75. 10.1111/jopy.12020 23126539

[63] Kurtz JE, Tiegreen SB. Matters of Conscience and Conscientiousness: The Place of Ego Development in the Five-Factor Model. Journal of Personality Assessment. 2005 12;85(3):312–7. 10.1207/s15327752jpa8503_07 16318570

[64] Bucher MA, Samuel DB. Development of a Short Form of the Abridged Big Five-Dimensional Circumplex Model to Aid with the Organization of Personality Traits. Journal of Personality Assessment. 2019 1 2;101(1):16–24. 10.1080/00223891.2017.1413382 29388839

[65] DeYoung CG. The neuromodulator of exploration: A unifying theory of the role of dopamine in personality. Front Hum Neurosci [Internet]. 2013 [cited 2020 Jun 10];7. Available from: http://journal.frontiersin.org/article/10.3389/fnhum.2013.00762/abstract 10.3389/fnhum.2013.00762 PMC3827581 24294198

[66] Hallquist MN, Wright AGC, Molenaar PCM. Problems with Centrality Measures in Psychopathology Symptom Networks: Why Network Psychometrics Cannot Escape Psychometric Theory. Multivariate Behavioral Research. 2019 8 12;1–25. 10.1080/00273171.2019.1640103 PMC7012663 31401872

[67] Thalmayer AG, Saucier G. The Questionnaire Big Six in 26 Nations: Developing Cross-Culturally Applicable Big Six, Big Five and Big Two Inventories: The Questionnaire Big Six in 26 nations. Eur J Pers. 2014 9;28(5):482–96.

[68] Soto CJ, Tackett JL. Personality Traits in Childhood and Adolescence: Structure, Development, and Outcomes. Curr Dir Psychol Sci. 2015 10 1;24(5):358–62.

[69] Condon DM, Roney E, Revelle W. A SAPA Project Update: On the Structure of phrased Self-Report Personality Items. Journal of Open Psychology Data. 2017 6 9;5:3.

[70] Roberts BW, Walton KE, Viechtbauer W. Patterns of mean-level change in personality traits across the life course: A meta-analysis of longitudinal studies. Psychological Bulletin. 2006;132(1):1–25. 10.1037/0033-2909.132.1.1 16435954

[71] Schwaba T, Robins RW, Grijalva E, Bleidorn W. Does Openness to Experience matter in love and work? Domain, facet, and developmental evidence from a 24‐year longitudinal study. J Pers. 2019 10;87(5):1074–92. 10.1111/jopy.12458 30693507

[72] DeYoung CG. Cybernetic Big Five Theory. Journal of Research in Personality. 2015 6 1;56:33–58.

